# Filling a hole in ozone research: The impacts of early life microbiome alterations on pulmonary responses to a non‐atopic asthma trigger

**DOI:** 10.14814/phy2.14346

**Published:** 2020-01-21

**Authors:** Richard A. Johnston, Peter Belenky

**Affiliations:** ^1^ Pathology and Physiology Research Branch Health Effects Laboratory Division National Institute for Occupational Safety and Health Centers for Disease Control and Prevention Morgantown West Virginia; ^2^ Department of Molecular Microbiology and Immunology Brown University Providence Rhode Island

The predominantly commensal collection of bacteria, fungi, archaea, protozoa, and viruses that inhabit multicellular organisms constitutes the microbiota, and their DNA is referred to as the microbiome. Early‐life microbiome perturbation influences the development of asthma (Russell et al., (2012)), which is a chronic lung disease that is characterized, in part, by persistent lung inflammation, cough, dyspnea, wheeze, variable expiratory flow limitation, and airway hyperresponsiveness (AHR). As a heterogeneous lung disease, asthma materializes as a diverse number of clinical phenotypes that result from exposure to either atopic or nonatopic stimuli (Wenzel, 2012).

Russell et al. (2012) demonstrated that the magnitude of atopic lung inflammation induced by antigen (ovalbumin) sensitization and challenge in an animal model, which mimics features of atopic asthma in humans, is dependent upon the age when the gastrointestinal microbiota is perturbed. For example, administration of vancomycin, a glycopeptide antibiotic, to neonatal mice exacerbated antigen‐induced lung inflammation that was assessed by enumerating the number of bronchoalveolar lavage (BAL) eosinophils. However, dosing adult mice with vancomycin did not exacerbate lung inflammation. Russell *et al*. (2012) proposed that heterogeneity in antigen‐induced lung inflammation between vancomycin‐treated mice of different ages was associated with differences in the composition of the gut microbiota yet did not present any extensive data to provide a mechanism for this phenomenon.

The impact of early‐life microbiome alterations on AHR and lung inflammation induced by exposure to ozone (O_3_), an air pollutant and a nonatopic asthma trigger, have not been previously addressed. In this issue, Brown, Tashiro, Kasahara, Cho, & Shore (2019) report the effects of reshaping the microbiome in male and female weaning mice on O_3_‐induced changes in lung pathology in adulthood. Specifically, the early‐life microbiome was perturbed by cohousing weaning C57BL/6 mice that were purchased from two different vendors (The Jackson Laboratory and Taconic Farms), which are known to have distinct gut microbiota (Ivanov et al., 2009; Velazquez et al., 2019). Prior single‐ and cohousing experiments have concluded that despite highly similar genetic backgrounds, C57BL/6 mice from these two vendors have markedly dissimilar responses, including antibacterial and antifungal immunity (Ivanov et al., 2009; McAleer et al., 2016). These divergent responses were attributed, in part, to differences in the progression of a Th‐17 immune response by segmented filamentous bacteria that are present in Taconic but not Jackson mice (Ivanov et al., 2009; McAleer et al., 2016).

Brown *et al.* (2019) demonstrated that cohousing of weanling C57BL/6 mice from Jackson and Taconic largely abolished any vendor‐related differences in the microbiome, which the authors attributed to coprophagy. Furthermore, increases in the number of BAL neutrophils, a hallmark feature of O_3_‐induced lung inflammation, and O_3_‐induced increases in airway responsiveness in cohoused male mice were significantly reduced as compared to same‐housed mice. These results are in contrast to those of Russell *et al*. (2012) who reported that manipulating the early‐life microbiome of neonatal mice with vancomycin exacerbated lung inflammation in response to antigen, an atopic asthma trigger. Although the early‐life microbiome was altered in these studies *via* different mechanisms (antibiotic administration vs. cohousing), it is doubtful that this leads to opposing responses to atopic and nonatopic stimuli. In support of this thought, the investigators previously demonstrated that O_3_‐induced lung inflammation and AHR were also reduced in germ‐free mice or mice treated with antibiotics (Cho et al., 2018). Thus, opposing responses of early‐life microbiome perturbations on lung inflammation induced by atopic and nonatopic stimuli may be dependent on the branch of the immune system that drives these responses. For example, O_3_‐induced lung inflammation is driven by the innate immune system while antigen‐induced lung inflammation is initiated and perpetuated by both the innate and the adaptive immune system.

Although no in‐depth mechanistic studies were presented by Brown *et al*. (2019) to clarify their observations, they do speculate that early‐life microbiome perturbation may alter the relative abundance of bacteria that generate short‐chain fatty acids (SCFAs). Consistent with this possibility, the authors report that members of the Firmicutes phylum, which are potent generators of SCFAs (Morrison & Preston, 2016), were more abundant in same‐housed Taconic as compared to same‐housed Jackson mice, yet the relative abundance of Firmicutes phylum members were decreased after co‐housing. This is particularly relevant as this same investigative team reported that SCFAs facilitate the development of O_3_‐induced AHR (Cho et al., 2018). Although the authors did not measure circulating levels of SCFAs, it is entirely plausible that decreases in the relative abundance of bacteria that generate SCFAs in co‐housed mice lead to less severe O_3_‐induced lung pathology. To confirm this, SCFAs (e.g. propionate and tributyrin) could be administered to co‐housed mice to determine if this restores the degree of O_3_‐induced AHR and lung inflammation to that observed in same‐housed mice. In addition, sequencing techniques with the ability to more precisely identify bacterial species and strains could be used to determine the relative abundance of SCFA producers within the two housing groups.

A key strength of this study is the inclusion of both male and female mice. This is appropriate as pulmonary responses to O_3_ are greater in male as compared to female mice (Cho et al., 2019). Furthermore, these divergent responses to O_3_ were associated with sex‐related differences in the composition of the microbiome and response to exogenous SCFAs. Dramatic sex‐related differences exist in the composition of the microbiome and levels of SCFAs in colonic contents from mice (Gao et al., 2019), which could provide a further connection between the observed microbiome‐related modulation and the previously observed sex dependence in O_3_ sensitivity (Cho et al., 2019).

In conclusion, Brown *et al*. (2019) confirm that early‐life microbiome perturbation alters the development of asthma, which further strengthens the existence of a “gut‐lung” axis. More importantly, the authors demonstrate that the effect of early‐life microbiome perturbation on lung pathology induced by a nonatopic asthma trigger (O_3_) discriminates based on sex and is also completely counter to that observed in response to an atopic asthma trigger. Finally, if these data are translatable to humans, the effect of potential new asthma therapeutics on the gut microbiome must be considered as we are now aware that the gut and lung communicate through an exquisitely elaborate system.

## CONFLICT OF INTEREST

No conflicts of interest, financial or otherwise, are declared by the authors.

## DISCLAIMER

The content contained within this publication is solely the responsibility of the authors and does not necessarily represent the official views of the Centers for Disease Control and Prevention or the National Institutes of Health.
